# The Effectiveness of an Intervention to Enhance Cooperation Between Sick-Listed Employees and Their Supervisors (COSS)

**DOI:** 10.1007/s10926-015-9606-1

**Published:** 2015-09-19

**Authors:** Nicole Hoefsmit, Inge Houkes, Nicolle Boumans, Cindy Noben, Bjorn Winkens, Frans J. N. Nijhuis

**Affiliations:** Department of Social Medicine, Faculty of Health, Medicine and Life Sciences, Caphri School for Public Health and Primary Care, Maastricht University, P.O. Box 616, Maastricht, The Netherlands; Department of Health Services Research, Faculty of Health, Medicine and Life Sciences, Caphri School for Public Health and Primary Care, Maastricht University, P.O. Box 616, Maastricht, The Netherlands; Department of Methodology and Statistics, Faculty of Health, Medicine and Life Sciences, Caphri School for Public Health and Primary Care, Maastricht University, P.O. Box 616, Maastricht, The Netherlands; Department of Work and Social Psychology, Faculty of Psychology and Neuroscience, Maastricht University, P.O. Box 616, Maastricht, The Netherlands

**Keywords:** Return-to-work, Sick leave, Effect evaluation, Intervention, Cooperation

## Abstract

*Introduction* Early return-to-work (RTW) after sick leave is considered to support employees’ quality of life. Successful RTW requires adequate cooperation between absent employees and their supervisors. This study assesses the effectiveness of an intervention for COoperation regarding RTW between Sick-listed employees and their Supervisors (COSS; i.e. ‘conversation roadmap’, monitoring of cooperation and, if necessary, extra occupational physician support). *Methods* In this field study, employees on sick leave for 2–10 weeks, aged 18 up to and including 60, and performing paid labour for at least 12 h per week were included. Terminally ill were excluded. Multivariate regression (correcting for baseline quality of life) was used to compare 6-months follow up data regarding quality of life between the groups. Using Cox regression analyses, time until first-, full-, and sustainable RTW was compared between groups. *Results* In total 64 employees received COSS or common practice. No significant group differences were found regarding all study outcomes. The COSS group had a higher chance of work resumption than the common practice group. The hazard ratio was 1.39 for first RTW (95 % CI 0.81–2.37), 1.12 for full RTW (95 % CI 0.65–1.93) and 1.10 for sustainable RTW (95 % CI 0.63–1.95). *Conclusions* COSS has no significant effects. Yet, the results regarding work resumption show a tendency towards effectiveness. Therefore, COSS can be further developed and applied in practice. Researchers should try to prevent some limitations of the present study in future research, for instance by finding a more common research setting.

## Introduction

Early return-to-work (RTW) after sickness absence is considered to be important for employees’ health and quality of life [1]. Also, employers benefit from early return to work, particularly financially, i.e. lower costs for productivity loss, replacement and guidance of sick employees.

Despite the benefits for both parties, many employees do not return to work early. Studies have indicated that among other things, bottlenecks in the cooperation between absent employees and their employers hamper early RTW (see, for example [2–4]).

On three different institutional levels [5], researchers and policy makers have developed initiatives to facilitate cooperation. Examples are national legislation (e.g. in the Netherlands, [6]), regional or local policy (in Canada, [7, 8]), or interventions for individual employees. An example of the latter is the workplace intervention by Karlson et al. [9, 10] to support communication between the employee and the supervisor, which successfully enhanced RTW at 1.5 year follow up for all participants and at 2.5 year follow up (the latter only for younger participants). Such interventions are typically developed for employees with specific health complaints (e.g. low back pain) and accessible through healthcare providers or insurers.

To address the bottlenecks in cooperation regarding RTW, we developed a generic (developed for all absent employees, regardless of their diagnosis) intervention that is provided at an organisational level. To the authors’ knowledge, such an intervention has not yet been evaluated. A strong need for a generic workplace intervention exists since it can be applied organisation-wide, for all absent employees, even without knowing their diagnoses (note that for example, Dutch legislation does not allow supervisors to ask employees for their medical diagnosis). Our intervention is entitled ‘COoperation regarding return-to-work between Sick-listed employees and their Supervisors’ (COSS). The intervention consists of A) a ‘conversation roadmap’ for employees and supervisors to structure and intensify their cooperation regarding RTW. This roadmap covers guidelines for sick-listed employees and their supervisors regarding which topics to discuss, as well as when and how this can be done. The intervention also contains B) regular monitoring of the quality of the cooperation between employee and supervisor and, if necessary, C) special support by an occupational physician (OP) to facilitate cooperation, based on the results of the monitoring. This monitoring occurs by means of an instrument (questionnaire) that measures several possible bottlenecks in the cooperation between absent employees and their supervisors (i.e. a lack of mutual trust and symbiotic dependency as well as open communication, planned- and time contingent approach of meetings and shared decision-making about RTW). These bottlenecks were found in earlier studies [11–13]. Employees and their supervisors both filled out the measurement instrument every few weeks (intervals varied with sick leave duration, the maximum interval was 12 weeks) until full RTW. OPs received a written report every time an employee and supervisor filled out the monitoring instrument. Prior to the start of COSS, participating OPs received training in supporting the cooperation between sick-listed employees and their supervisors. Yet, they were free to decide about whether and how they actually supported the cooperation between sick-listed employees and their supervisors. The development, the process evaluation and the economic evaluation of COSS are described elsewhere [14, 15].

The present study evaluates the effectiveness of COSS, which is especially relevant for RTW professionals who aim to develop effective interventions in the Netherlands and other Western countries. The aim of this study is to detect whether COSS achieves better results concerning quality of life, first RTW (time until first progress made in working hours), full RTW (time until complete work resumption), and sustainable RTW (time until lasting complete RTW, i.e. working for 4 weeks without relapse in partial or complete sick leave) when compared to common practice.

## Methods

### Design and Setting

A field study was performed in a large Dutch banking organisation. We aimed to cluster randomise at department level. However, due to practical reasons our control group consisted of only one cluster and we chose to ignore the cluster randomisation in our analyses. According to Dutch law, our study did not require ethical committee approval (correspondence dd. 7 November 2011, registration number: METC 11-4-115/Dutch trial register: 3151).

### Participants

Inclusion criteria for employees were that they had to be:

- on sick leave for at least 2 weeks but no longer than 10 weeks;
- aged from 18 up to and including 60 years; and
- performing paid labour for at least 12 h per week.

Those who were terminally ill were excluded.

The criteria assessment was part of the baseline questionnaire. After approximately 5 weeks of sick leave on average, employees and their supervisors were included in either the common practice group or the COSS group, which received the intervention.

**Fig. 1:**
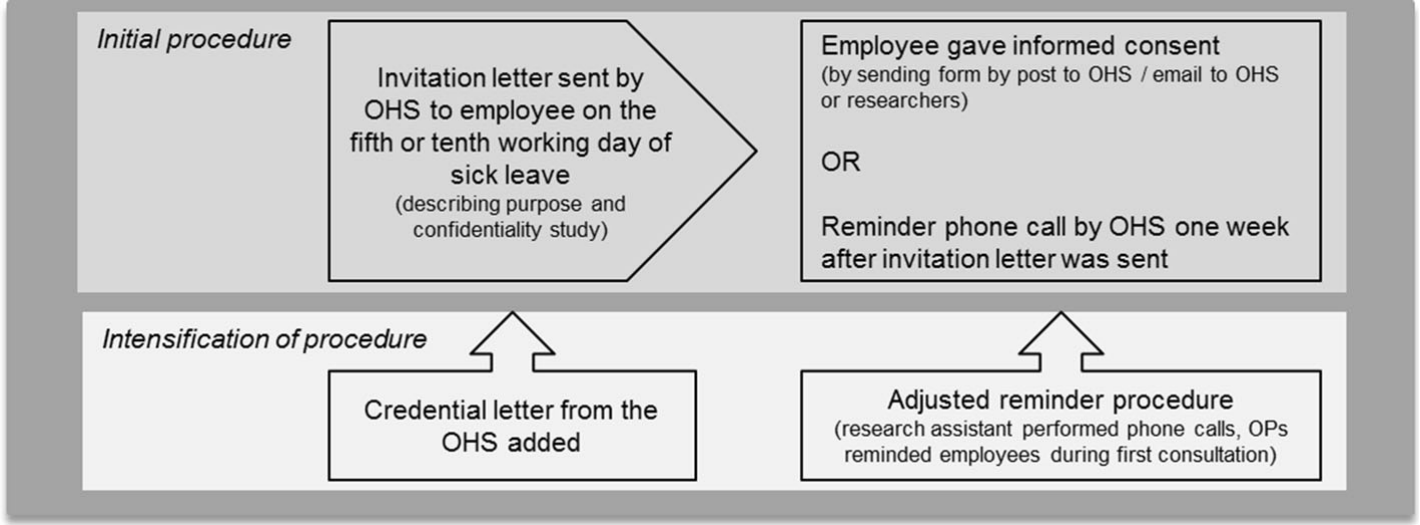
Recruitment procedure. *OHS* occupational health service

Inclusion took place between April 2012 and December 2013. Potential study participants were selected from all sick-leave cases at the participating organisation on the fifth or tenth working day of their sick leave. Figure 1 describes the recruitment procedures.

Our initial recruitment strategy did not yield a sufficient number of participants within the time span available. Therefore, the recruitment strategy was modified by intensification and adding more endorsement from the organisation (see Fig. 1). A considerable part of the invited employees already returned to work, were about to resume work or were not sick-listed in the first place. Prior to the study all participants received information about the purpose and procedure of the study and all participants gave their informed consent for participation.

Sample size calculation indicated that at least 60 employees per group (COSS group and common practice group) were needed. Taking into account a dropout rate of 15 %, about 70 employees in each group were required. However, this calculation was based on assumptions that were not completely correct. With N = 60 employees per group, a Cohen’s *d* of 0.6 can be detected, which is a medium to large effect size [16].

### COSS and Common Practice

Table 1 describes the support during sick leave and RTW prescribed for the COSS and common practice group.

**Table 1:** COSS and common practice

Common practice		What COSS adds to common practice
Legislation [6, 14]	Organisational policy in addition to legislation	
Legislation prescribes several minimum requirements of cooperation between absent employee and employer such as writing action plan for RTW and regular evaluation of its progress	First day sick leave: Telephonic contact employee-supervisor on first day	Conversation roadmap (step by step plan in booklet format) to structure and intensify cooperation employee- supervisor
Employee compensated by employer (≥70 % income)	Week 2–3 sick leave: Employee and supervisor fill out form about, among others, estimated sick leave duration. Employee who is unsure about the estimated sick leave duration or reports psychological complaints, is invited by OP	Monitoring quality of cooperation (employee and supervisor fill out questionnaires). Every 4–12 weeks, research team analyses results using cut-off scores
	Throughout process: weekly meetings employee-supervisor	If necessary, based on questionnaire results, extra support of cooperation provided by OP

In both groups, the RTW process should comply with legislation. Also, employees received support based on the organisation’s (not obligatory) policy. Additionally, the intervention group received COSS, which is described in more depth elsewhere [14]

*OHS* occupational health service, *OP* occupational physician

Both in the COSS and common practice group, the RTW process should comply with Dutch legislation. Moreover, employees received support based on the organisation’s own (not obligatory) sick leave policy. Additionally, the intervention group also received COSS.

### Study Variables and Data Collection

Outcome measures of this effect evaluation were employees’ quality of life, first-, full-, and sustainable RTW. Quality of life was assessed measuring self-reported outcomes on five domains (i.e. mobility, self-care, usual activities, pain/discomfort and anxiety/depression) using the validated EuroQol 5 Dimensions 5 Levels (EQ-5D-5L, response range: 1–5) [17, 18]. Both in the COSS group and common practice group, employees filled out questionnaires at baseline and at 6 months follow up. First RTW was operationalized as the time in calendar days from the first sick leave day until the first progress made in working hours. Full RTW was the time in calendar days of sick leave until complete work resumption. Sustainable RTW was the time in calendar days of sick leave until lasting complete work resumption (working for 4 weeks without relapse in partial or complete sick leave). We used data of the organisation’s sick-leave administration for measuring the period between the start of the sick leave period wherein the employee started to participate in the study until first RTW, full RTW and sustainable RTW. This concerned a period somewhere between 23 April 2012 up to and including 7 January 2014. This implies that the follow up duration of the work resumption data varied between employees.

Additionally, by means of the baseline questionnaire, information on general characteristics was collected: education, age, gender, caring for children below 12 years of age and working hours per week.

All self-reported questionnaires were filled out electronically. When participants did not respond within 1 week after invitation, weekly reminders were sent by email. When participants did not respond to the reminders, they received a phone call by the university’s research assistant.

**Fig. 2:**
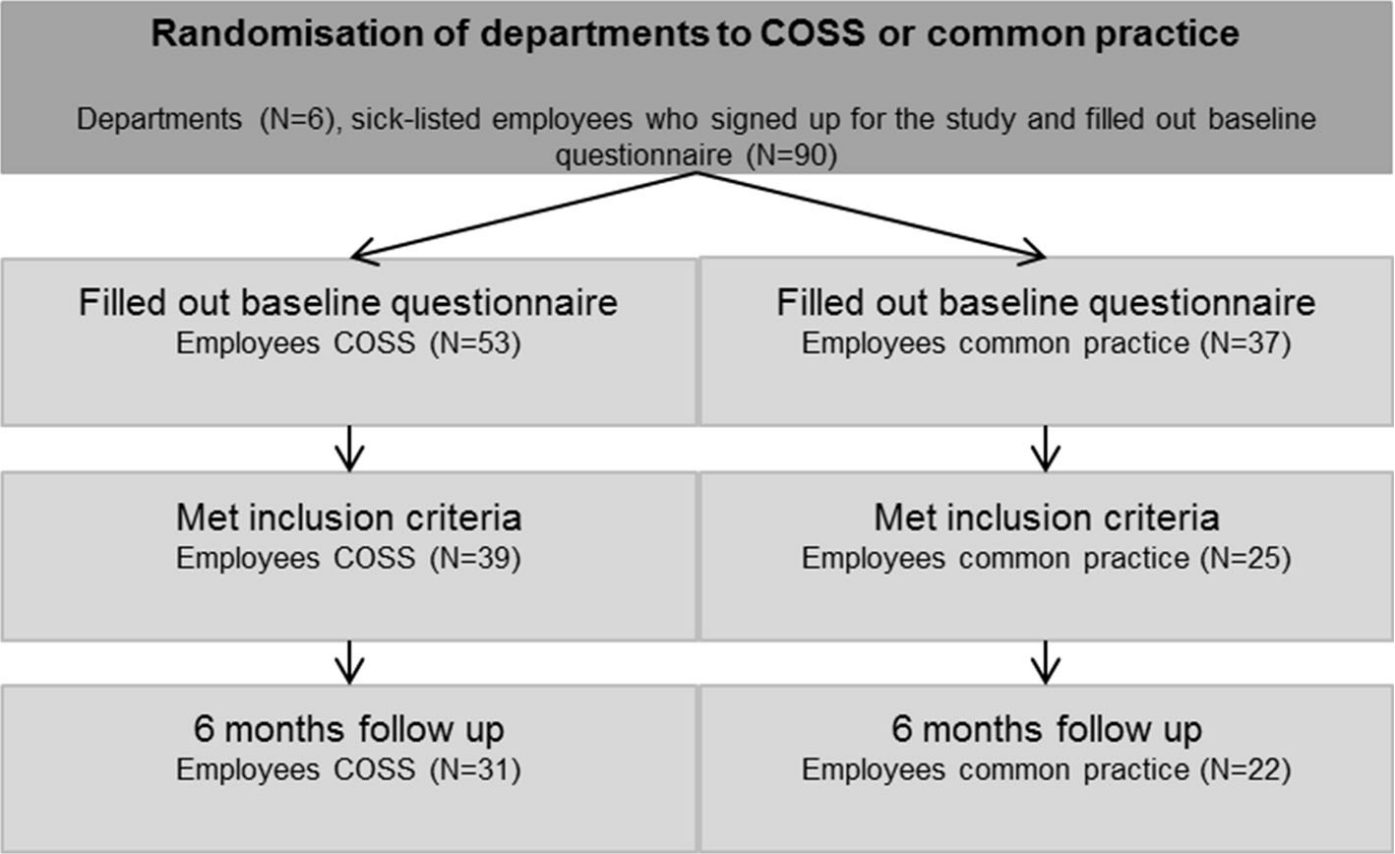
Study sample. Cooperation between sick-listed employees and their supervisors (COSS)

Figure 2 shows the inclusion flow of the study participants.

In total 64 employees were included and analysed; 39 in the COSS group and 25 in the common practice group.

### Analyses

Intention-to-treat analyses (unit of analysis: employee) were performed. Quality of life index values were calculated using the EQ-5D-5L Crosswalk value set [19]. The mean score was imputed in case of missing quality of life index values and the mode was imputed where respondents indicated that their education was ‘other’. In case education is bimodal, the mode score that is closest to the median will be used.

For the baseline participant characteristics, numerical variables were presented by mean (SD) and categorical ones by number (%).Group differences in the numerical and categorical variable were tested using independent-samples *t* test and χ^2^ test, respectively.

Next, we presented the mean (SD) regarding the follow up of quality of life for the COSS- and common practice group separately. Linear regression analysis, with correction for baseline quality of life, was performed for the outcome quality of life at follow up.

Survival analyses (Kaplan–Meier curves and Cox regression) were performed for the outcomes first RTW, full RTW and sustainable RTW. In the Cox regression, we tested the proportional hazards assumption by adding a time dependent covariate (interaction of group with time) to the model which included only group.

Sensitivity analyses were also performed. First, we repeated the linear regression analysis for the outcome quality of life in a dataset without imputations of missing quality of life index scores. Then, for both quality of life and the work resumption outcomes, we corrected for participants’ characteristics that differed significantly between the COSS- and common practice group at baseline. Due to small group sizes, we added these characteristics separately to the linear- (with outcome quality of life) and Cox regression model (with outcomes first RTW, full RTW, sustainable RTW).

All analyses were performed using IBM SPSS Statistics for Windows, Version 22.0 (SPSS, 2013 ,New York, USA) and significance was set at a two-sided *p* ≤ 0.05.

## Results

### Participant Characteristics

Table 2 describes characteristics of the final study sample.

**Table 2:** Characteristics of the study participants in the study groups

Variable	COSS group (N = 39)	Common practice group (N = 25)	*p* value
Education, N (%)			
Low	5 (12.82)	3 (12.00)	0.12
Intermediate	22 (56.41)	8 (32.00)	
High	12 (30.77)	14 (56.00)	
Age, mean (SD)*	45.31 (9.17)	50.60 (7.44)	0.02
Gender, N (%)*			0.01
Male	14 (35.90)	18 (72.00)	
Female	25 (64.10)	7 (28.00)	
Taking care of children <12 years, N (%)	18 (46.15)	6 (24.00)	0.07
Working hours per week, mean (SD)*	31.72 (6.83)	35.20 (4.59)	0.02
Baseline index value quality of life, mean (SD)	0.65 (0.16)	0.63 (0.24)	0.81

Low education covers lower professional education, middle secondary general education. Intermediate education consists of apprenticeship or short middle professional education as well as middle professional education and secondary general education. High education covers higher professional education and academic education

* *p* ≤ 0.05

The table shows that, compared to the COSS group, the common practice group was significantly older, consisted of significantly more males and worked significantly more hours per week.

### Quality of Life

Table 2 shows that at baseline, the mean quality of life was 0.65 (SD = 0.16) in the COSS group and 0.63 (SD = 0.24) in the common practice group. At follow up, the mean quality of life index value was 0.81 (SD = 0.10) in the COSS group and 0.83 (SD = 0.10) in the common practice group. After correction for baseline in the multivariate regression analyses, there was no significant group difference (corrected mean difference: −0.02, 95 % CI −0.07 to 0.03). The sensitivity analyses did not yield substantially different results.

### Work Resumption

**Fig. 3:**
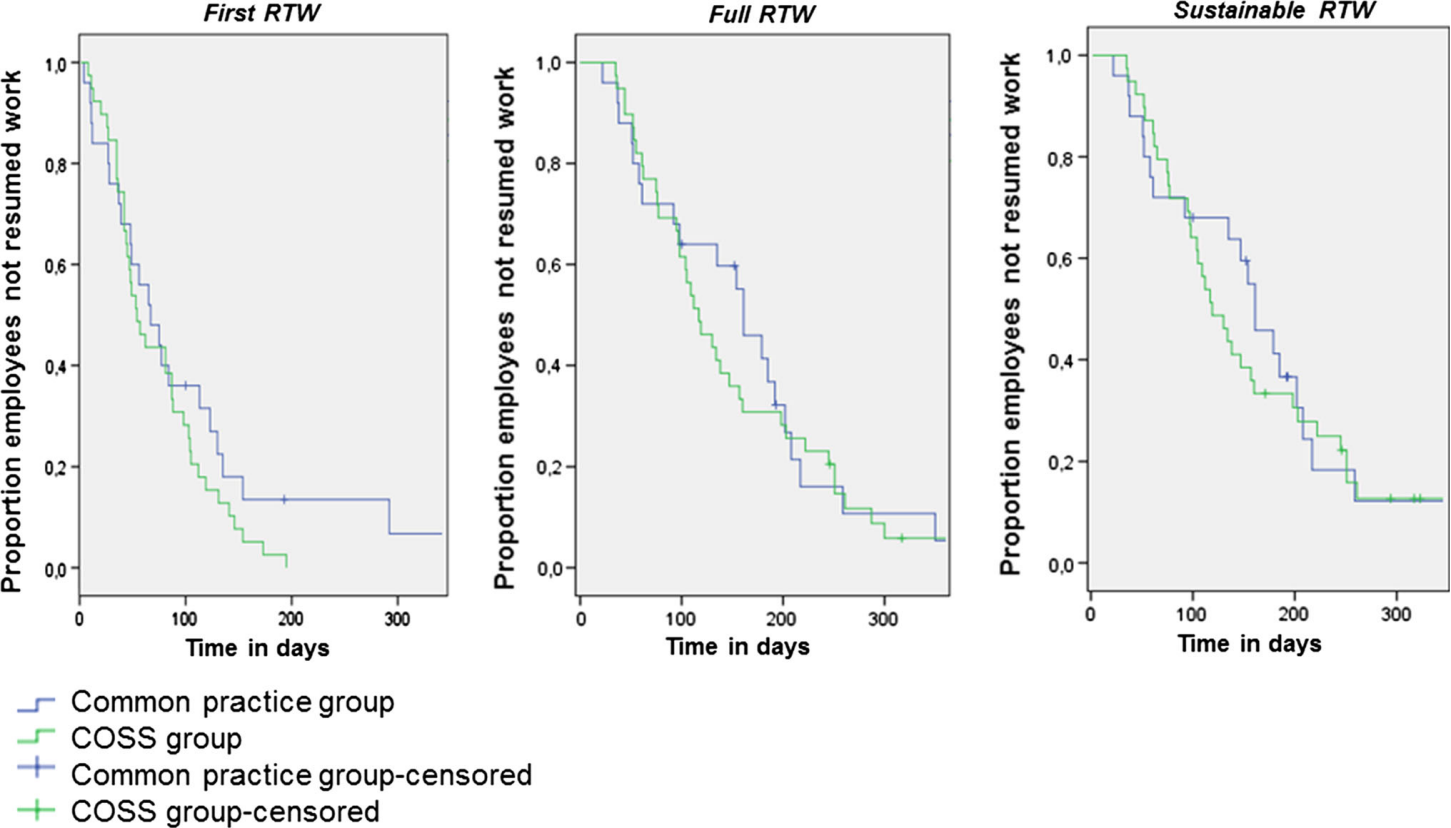
Kaplan–Meier curves for first RTW, full RTW and sustainable RTW

Figure 3 shows the Kaplan–Meier curves for the COSS- and common practice group regarding first RTW, full RTW and sustainable RTW.

The curves show that between about 50 and 100 days as well as after 100 days (first RTW) and between about 100 and 200 days (full RTW, sustainable RTW) there was a smaller proportion of the employees in the COSS group on sick leave compared to the common practice group.

By means of Cox regression, we tested the proportional hazards assumption (i.e. interaction of group with time). This variable was not significant (first RTW, *p* = 0.13/full RTW, *p* = 0.70/sustainable RTW, *p* = 0.69). Therefore, we did not include it in the final model. Cox regression analyses showed that, although not significant, the COSS group had a higher chance of work resumption than the common practice group. The hazard ratio was 1.39 for first RTW (95 % CI 0.81–2.37, *p* = 0.23), 1.12 for full RTW (95 % CI 0.65–1.93, *p* = 0.68) and 1.10 for sustainable RTW (95 % CI 0.63–1.95, *p* = 0.73). Finally, the sensitivity analyses regarding work resumption did not yield substantially different results.

## Discussion

The aim of this study was to evaluate the effectiveness of COSS on quality of life, first RTW, full RTW and sustainable RTW. Although no significant effects were found, the results show a trend towards a positive effect on the work resumption outcomes.

The design of this field study was of good methodological quality, i.e. validated instrument to measure quality of life, multiple outcome measures for work resumption, objective sick leave data and advanced statistical methods. Yet, research in practice settings is complex and therefore the effect evaluation of COSS was carried out somewhat differently than planned. In the end, both unforeseen methodological factors and factors related to the content and implementation of COSS help to understand the lack of significant intervention effects. Yet, the lack of statistical power appears to be the main issue.

### Methodological Explanations for the Lack of Significant Effects

First, although we tried multiple strategies to recruit sufficient study participants, there is a lack of statistical power. It might be assumed that most people return to work smoothly, and that perhaps 20 % of the employees need a more intensive intervention than is provided in the common practice condition. In this case a sample size of 200–300 participants is required to see statistically significant results. A limited statistical power implies a reduced ability to find true/significant relationships between concepts [20].

Second, the results of the process evaluation of COSS showed that employees and supervisors generally were satisfied with their OP [14], suggesting that common practice is already of good quality. In line with this, selection effects may have played a role. Particularly those employees and supervisors who already had a satisfactory working relation before the onset of the employee’s sick leave may have been inclined to try COSS, Their adequate contact may have made them feel more comfortable to jointly try something new as COSS. Moreover, particularly employees with a high motivation to resume work may have been intended to start with COSS. Also, at the moment, sick leave in the Netherlands is at the lowest level since the year 1996 [21]. This may relate to the current economic crisis.

The study limitations described above mean that COSS is tested in a not advantageous setting that very likely entails an underestimation of the actual intervention effect. This results in a very limited chance for the intervention proven to be significantly effective.

### Explanations Related to the Content and Implementation of COSS

First, there were issues related to the content of COSS. A process evaluation indicated that a questionnaire was not an adequate tool to monitor the quality of the cooperation between employees and supervisors. Also, COSS would be particularly useful in situations characterised by uncertainty, e.g. an unclear medical prognosis or in case contact between sick-listed employees and their supervisors does not come about spontaneously [14]. This finding is in contrast with our expectation that generic interventions would be most useful. To our knowledge, COSS is rather unique, which complicates the possibilities for a thorough comparison with international literature. Yet, our finding is in line with the finding regarding a similar intervention as described by Karlson et al. [9, 10] which was successful among employees with a burnout. Such a group of employees may also experience an uncertain situation regarding their iterative (rather than a linear) process of medical recovery.

Second, there was a limited implementation of COSS. The intervention was often not used during the first weeks of sick leave (i.e. the conversation roadmap was distributed by e-mail to employees and their supervisors after approximately 5 weeks of sick leave on average). Also, the process evaluation of COSS revealed a limited use of COSS later during sick leave as well, which may relate to our process evaluation finding that COSS was considered to be useful in uncertain situations primarily [14]. The limited use of COSS may have undermined the effectiveness of COSS in this evaluation.

Overall, the methodological-, intervention- and implementation related factors described above complicate the possibilities to interpret the exact effect of COSS on the outcomes measured.

## Conclusions and Recommendations

This project is a further step in the study of organisational interventions to support cooperation between sick-listed employees and their supervisors in a generic population. We designed a field study of an overall high methodological quality and found no significant intervention effects. Yet, the results showed a tendency towards intervention effectiveness regarding the work resumption outcomes. The lack of significant effects was attributed to methodological limitations (e.g. limited power), COSS-related limitations (e.g. questionnaire was not an adequate monitoring instrument) and COSS was only partially used.

RTW professionals can adjust COSS to make it a generic intervention that can best be applied in uncertain situations such as when contact between sick-listed employees and their supervisors does not come about spontaneously. More complete intervention may be needed to deal with this uncertainty. For example, in further developments COSS should provide employees and supervisors with more concrete tools for work modification. In addition, future versions of COSS should also support a more intensive alignment and cooperation between the employees and the occupational physician with other stakeholders in the employees’ sick leave such as the general practitioner and other physicians. Recommendations regarding the implementation of COSS are provided elsewhere [14].

Researchers could try to prevent some limitations of the present study in future research, for example by selecting a more common research setting, as can for instance be found in organisations with less outstanding and more usual quality of common practice (i.e. many Dutch organisations have a general sick leave policy only and supervisors naturally undertake less effort themselves as they rely more on the OP support in RTW guidance).

## Abbreviations

COSS: Cooperation between Sick-listed employee and supervisor
METC: Medical ethical committee
OHS: Occupational health service
OP: Occupational physician
RTW: Return-to-work
